# Editorial Notes

**Published:** 1921-06

**Authors:** 


### Editorial Notes.

---

**Solving the Financial Problem of our Voluntary Hospitals.**

THE annual meetings of the Governors of the Bristol Royal Infirmary and Bristol General Hospital held in May adopted the new regulations recommended by the joint sub-committee of their General Committees, viz. that subscribers' notes should be abolished and that in-patients should contribute a maximum of one guinea a week towards the cost of their maintenance, provided their means allowed such a contribution to be made ; from necessitous patients no contribution would be asked for. Furthermore, a contribution of sixpence per attendance would be collected from out-patients and some contribution sought from those who had to be treated by massage or in the electrical department.

Each of these Hospitals, moreover, resolved to institute an almoner under the jurisdiction of their House Committee.

One cannot allow the occasion to pass without placing on record the munificent gift of Mr. H. H. Wills, the President of the Bristol Royal Infirmary, in presenting securities to the value of £105,000 to this institution. That it helps very much towards meeting a serious difficulty is obvious, the gift so speaks for itself as to require no comment ; but what is not obvious to those who have not been associated in this and other good work that Mr. Harry Wills puts his hand to is the devotion, time and energy he gives personally towards promoting the interests and the continued success of the Royal Infirmary.

**Bristol Royal Infirmary's Honorary Medical Staff's Offer.**

THE Medical Faculty of the Royal Infirmary made a definite offer to the Governors of the Royal Infirmary which it is well to place on record, as it indicates the desire of the Honorary Staff to perpetuate and promote the Voluntary Hospital system on the soundest basis possible :—

" In order to enable the Governors to bring into operation the proposals for the admission of paying patients, the Honorary Staff desire to offer their gratuitous services to all patients who are found, after inquiry by the almoner, to be unable to pay more than the cost of their maintenance. But in the case of patients whose payments are defrayed by public authorities or approved societies the Honorary Staff will attend and treat such patients in consideration of an agreed percentage of the gross payment received by the Royal Infirmary being allocated to and paid into a fund which shall be administered by the Honorary Staff."

**The Hospital Almoners' Council Report.**

THIS annual report brings home the valuable adjunct to the work of hospitals that is being furnished by the trained almoner. The Council emphasise the need for special training as well as a liberal education, and furthermore they lay stress on the innate sympathy and tactful disposition which are essential qualifications in those who are to engage in this responsible work. As regards the difficult question of patients' contributions, the Council's report is worth citing, being based on experience :—

" The point still unsolved is, how can the greatest sum be raised with the least suffering to the patients and without disturbing the old happy relationship which has existed between the voluntary hospital and the community which it serves. There are two methods by which sums from this quarter can be raised. One is by the establishment of a fixed scale or flat rate of payment for in-patients and out-patients per week or per attendance according to age and sex. This method is advantageous in that it insures a clear estimate of the sum possible to raise. It involves a danger, however, in that it must give rise to a reluctance to attend a hospital on the part of those patients who can ill afford the fee until they are compelled to do so by the severity of their illness. There are also a number of patients attending voluntary hospitals who, although unable to pay the fees of a specialist and nursing home or even of a general practitioner for a long period of treatment, can well afford to pay a sum larger than the rate fixed by the hospital, which must necessarily be low in comparison to the cost of treatment. Under this system the majority of people will probably be prepared to pay the fixed scale, but a considerable minority will be either too poor to attend at all, or will be able to make payment only at the cost of foregoing some serious necessity of life, or they will be so well-to-do that they should contribute a much higher proportion to the cost of treatment. The second method is that of inviting voluntary contributions from patients according to their means through the direct medium of the almoner. To justify an almoner's department from a financial aspect is not altogether easy until it is realised that much of her work lies in the co-ordination of intelligent understanding with the science of healthy living. Many hospitals are finding the solution to the problem by creating such a department—not only to raise money justly and considerately, through genuine knowledge of social conditions, but also to relieve the cases of necessity which inquiry brings to light and so to complete and solidify the work of the hospital."

The constitution of an almoner at each of the Bristol voluntary hospitals is a great step towards protecting the subscribers from misuse of their donations, the poorer patient from being crowded out by the relatively well-to-do, and the medical staff from an unfair exploitation of their honorary services which the vast majority of the British workers would be the first to resent. But it seems a pity that the institution of a conjoint almoner department should have been postponed, the advantages of such a measure, which has nothing but advantage in its favour, has not yet been brought about. Common sense and economy will surely ensure amalgamation of the almoner departments in the near future.

**Medical Postgraduate work in the University of Bristol.**

The series of weekly clinical demonstrations arranged by the Postgraduate Committee of the Clinical Board, which commenced on May 11th, have proved most successful. The number of practitioners who joined the course exceeds that of 1920, and the weekly attendance has been higher than on any previous occasion. Many of those attending have also joined as clinical assistants in various departments of hospital work, and it seems probable that this method of study will become increasingly popular. It may be remembered that a course of Clinical Pathology and Bacteriology was held in 1910 at Taunton, Frome, Dorchester, and other towns, and it has now been arranged. at the request of the local practitioners, to give a course of clinical lectures and demonstrations, in the ensuing autumn, at Trowbridge, a centre which will permit the attendance of medical men from all parts of Wiltshire. We believe that no other University has made the experiment of giving postgraduate courses at country centres, and it is certain that the example set by Wiltshire will be watched with keen interest, and if successful copied by other districts. The increasing importance attached to postgraduate work is evidenced by the fact that a Committee of Inquiry has recently been appointed by the Ministry of Health to inquire into the need of postgraduate study both in London and the provinces. On March 11th the Chairman of the Bristol Committee attended in London and gave evidence on behalf of the University of Bristol.

**University of Bristol. Resignation of the Vice-Chancellor.**

Sir Isambard Owen's resignation of the Vice-Chancellorship of our University has been received with very deep regret not only by the Council, Academic Staff and the Undergraduates of the University but by the Citizens of Bristol and a very wide circle in the neighbouring counties. Sir Isambard's leaving will be one unfortunate outcome of the new rules of the Treasury with regard to superannuation which could not be made for those whose energies and mental alacrity appear unabated at an age when most of us claim release from office involving such multifarious responsibilities. A distinguished physician and lecturer on Medicine at St. George's Hospital, London, he took a prominent part in the movement leading to the establishment of the University of Wales. He relinquished his hospital work and his practice in 1904 when he was appointed Principal of Armstrong College, Newcastle-upon-Tyne. In 1909 he became Vice-Chancellor of our University, and we have had the benefit of the experience he gained in University organisation at Durham. No one could have been more successful in the organisation of our new University, and his genial bearing and dignified presence has drawn together the workers in every department of our University work and life. When the time of his departure from our midst arrives he will carry away the hearty good wishes of us all and the assurance that he and Lady Owen, who, like her distinguished husband, has endeared herself to the University, leave us with gratitude and admiration for their work here and with the hope that they have before them many years of useful work and of happiness in some different spheres of activity.

**J. M. Fortescue-Brickdale, M.A., M.D. Oxon., M.R.C.P.**

Our obituary of Dr. J. M. Fortescue-Brickdale records the good work of our late colleague and of the loss sustained by the medical profession in his death in the prime of life.

The Editorial Committee of *The Bristol Medico-Chirurgical Journal*, profoundly conscious of the loss the medical profession has sustained by the death of Dr. J. M. Fortescue-Brickdale, record their appreciation of his services as Editorial Secretary since his appointment in January, 1913. His exceptional scientific and literary attainments and untiring devotion to the interests of the *Journal* contributed very materially to its success. We mourn with a deep sense of personal bereavement a colleague whose lovable disposition endeared him to us, and tender heartfelt sympathy to his widow and all the members of his family.

Adieu, beloved colleague. To have known you and worked with you was truly a privilege; simple words may convey, but cannot measure, all we feel in losing you.

